# The Influence of Bullying on Positive Emotions and Their Effect as Mediators between Controllable Attributions of Success and Academic Performance

**DOI:** 10.3390/children10060929

**Published:** 2023-05-24

**Authors:** Antonio Ragusa, Valeria Caggiano, Ana Isabel Obregón-Cuesta, Jerónimo J. González-Bernal, Jessica Fernández-Solana, Luis Alberto Mínguez-Mínguez, Benito León-del-Barco, Santiago Mendo-Lázaro, Ema Di Petrillo, Josefa González-Santos

**Affiliations:** 1 Rome Business School, Department of Education, 00196 Rome, Italy; ragusa@romebusinessschool.it (A.R.); dipetrillo@romebusinessschool.it (E.D.P.); 2 Department of Education, University Roma TRE, 00154 Rome, Italy; valeria.caggiano@uniroma3.it; 3 Department of Mathematics and Computing, University of Burgos, 09001 Burgos, Spain; 4 Department of Health Sciences, University of Burgos, 09001 Burgos, Spain; jejavier@ubu.es (J.J.G.-B.); jfsolana@ubu.es (J.F.-S.); mjgonzalez@ubu.es (J.G.-S.); 5 Department of Education Sciences, University of Burgos, 09001 Burgos, Spain; laminguez@ubu.es; 6 Department of Psychology and Anthropology, University of Extremadura, 10071 Caceres, Spain; bleon@unex.es (B.L.-d.-B.); smendo@unex.es (S.M.-L.)

**Keywords:** positive emotions, internal controllable attributions, academic performance, exams, bullying, primary school, secondary school, educational context

## Abstract

Academic performance (AP) is a topic of particular interest in the academic context. Attributions for academic success (AAS) have been shown to have a significant impact on AP, and more specifically internal controllable attributions (ICA) are closely linked to academic success. Similarly, positive emotions (PE) have a significant influence on AP and may in turn be influenced by bullying. This study examines the connections between ICA of academic success and AP mediated through PE in late primary and early secondary school students and analyzes the relationships between PE and bullying categories. Students (N = 562, 49.46% female, M_age_ = 11.6 SD = 1.2) reported on their perceptions of ICA and PE in relation to exams and their relationship with bullying through validated questionnaires. The AP was obtained as the average mark of all subjects in the immediately preceding assessment. First, a multiple linear regression analysis considering ICA and PE as predictor variables was carried out, which showed a significantly positive link between ICA and PE, between ICA and AP, and between PE and AP. Subsequently, using the SPSS macro PROCESS, a simple mediation model was implemented to quantify the effect of ICA on AP through PE in exams, and finally an ANOVA between the categories of bullying and PE was performed. The results showed a significant indirect relationship with a positive predictive relationship for AP. The model shows that PE proves to be a significant mediator between ICA and AP, and it is shown that students disengaged from bullying score higher in PE.

## 1. Introduction

In the educational field, academic performance (AP) is a topic of special interest and the subject of considerable research. AP refers to a student’s performance in his or her studies and can be measurable through examinations and/or grades. It can be related to various factors, such as cognitive skills, motivation, effort, or student characteristics [1,2]. Some educational models go beyond individual non-cognitive factors that increase the predictability of AP, such as personal or contextual factors [3]. Motivation, personality traits, personal factors, emotions, and their involvement or not in bullying can be included as factors determining successful AP [4].

One variable that has been shown to have a significant impact on AP is attributions for academic success (AAS). This refers to the explanations that people give for their success in a particular event. These can also be internal, such as effort or innate characteristics/skills, or external, such as help from others or luck [5].

Weiner’s theory tries to understand how the causes of success are related to AP [6,7]. Each attributional style favors or hinders learning by determining the motivation with which students carry out academic tasks and influences their self-perception and AP [8]. Many studies have agreed that success is closely linked to internal controllable attributions (ICA) such as effort, hard work, or learning, i.e., factors that are internal and controllable by the individual [8,9,10].

Some studies have already shown that if students attribute their successes to their efforts or abilities, they are likely to feel proud and motivated to continue performing well; this has important implications for their academic growth [11,12]. The changes in children’s problem-solving attributions result from metacognitive developments that not only determine their emotional reactions but also their task orientation [13].

Thus, ICAs have a positive effect on AP, fostering motivation and self-regulation, leading to greater effort and thus better AP [3]. Moreover, when students attribute their AP to internal controllable factors, such as effort, they demonstrate higher self-efficacy and greater persistence in complex academic tasks [14]. This can lead to a greater sense of control and responsibility for one’s own success, leading to greater motivation and effort to achieve future goals [15,16].

Similarly, the role of emotions in the academic context is worth noting. In these educational contexts, students experience emotions that are related to learning in different pedagogical moments, such as performing an individual task or completing an exam [17,18]. Emotions directly linked to achievement activities are present in all teaching–learning processes and it is essential to understand them in order to maximize learning [19].

In this regard, positive emotions (PE) experienced by students have been shown to have a significant impact on their AP as well as their general well-being [20]. When students have these types of emotions, such as joy, gratification, or motivation, they have a greater capacity to learn and retain new information, a greater ability to cope with new challenges, and greater resilience. In addition, those who experience PE have higher intrinsic motivation, which translates into greater effort and better AP [21,22]. Some studies have shown that students who have greater emotional regulation tend to have better academic results and greater satisfaction with academic life compared to other students who have difficulties in regulating their emotions [23].

Thus, we have found two variables, PE and ICA of academic success, to have an important and significant influence on another variable, AP. However, to our knowledge, no studies have been published on how these three interact together. Mediation analysis is a statistical technique that determines whether a mediating variable, in this case PE, can interpose itself between two other variables, AP and ICA, to explain a relationship between them. It attempts to determine whether the relationship between an independent variable and a dependent variable is largely explained by the relationship between the independent variable and the mediating variable, and between the mediating variable and the dependent variable [24].

As a final point, it is worth noting the important role that bullying plays in PE and AP. Finally, it is worth highlighting the important role that bullying plays in positive emotions and academic performance. Additionally, bullying should be considered a social phenomenon that can have serious consequences, causing physical, psychiatric, and emotional symptoms, including low academic achievement. This is associated with a deterioration in quality of life and problems in social relationships [25,26,27,28,29,30]. Physical, verbal, and/or social abuse represents a significant health problem for students. Victims may experience worse emotional, social, academic, and health development, while aggressors often exhibit delinquent and aggressive behaviors later on [30,31]. It has also been demonstrated that engaging in bullying behaviors, both as a victim and as an aggressor, is associated with negative outcomes among students, ultimately resulting in school dropout. Greater involvement in bullying is associated with greater negative consequences for academic achievement [32,33]. Previous research has shown a strong relationship between PE and the different categories of bullying [34]. It has been observed that lower PE is related to bully/victim behaviors, while higher PE is demonstrated by students not involved in bullying. However, it is currently unknown whether there is a relationship between PE and the different categories of bullying, which in turn influence students’ AP.

**Figure 1:**
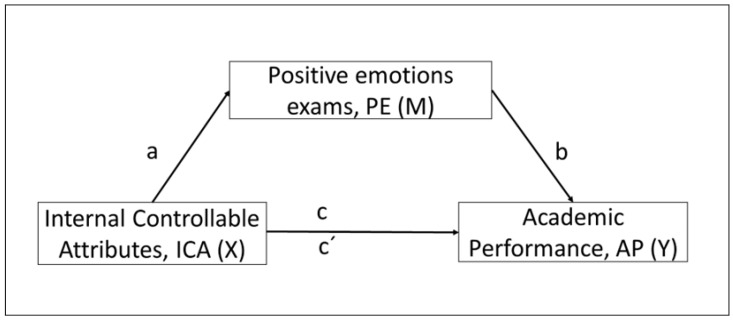
Diagram of the simple mediation model. Indirect effect of internal controllable attributions (ICA) to academic success on academic performance (AP) through positive emotions about exams (PE).

This study (Figure 1) aimed, on the one hand, to test a model in which the independent variable was ICA of academic success, the mediating variable was PE on exams, and the dependent variable was AP. More specifically, it sought to quantify the effect of ICA of academic success on performance through PE in relation to exams and, on the other hand, to demonstrate the existence of a relationship between the different categories of bullying and PE.

## 2. Materials and Methods

### 2.1. Participants

An ex post facto cross-sectional research design was followed to assess connections between variables without direct intervention. Participants were selected by means of stepwise cluster sampling in public and private schools in Castilla y León, located in urban areas.

The sample consisted of 562 students in five schools, public (n = 4) and private (n = 1), of Compulsory Primary Education (EPO) and Compulsory Secondary Education (ESO). The mean age was 11.66 years (SD = 1.2, range = 10–15). EPO students (n = 334) were in the fifth (n = 228) and sixth (n = 186) years, and ESO students (n = 148) were in the first (n = 134) and second (n = 94) years. Of these, 284 students (0.51%) were boys and 278 (0.49%) were girls.

### 2.2. Procedure

In accordance with the ethical guidelines set forth by the American Psychological Association regarding consent, confidentiality, and anonymity, a member of the research team reached out to school principals to inform them about the research objectives.

Although a total of 8 schools were contacted, only 5 of them agreed to participate in the research. The schools that declined cited time constraints in the classroom and diffi-culties in obtaining parental consent as reasons for non-participation.

Once the collaboration was approved, participants were approached in their class-rooms. After securing informed consent from their parents or legal guardians, the partici-pants proceeded to complete the scales. The completion of the scales was conducted anonymously to ensure the confidentiality of the collected data, which would be exclu-sively used for research purposes. The administration of the scales took place during school hours, with detailed instructions provided and any questions addressed during the process. Emphasis was placed on the anonymous nature of the investigation. The ques-tionnaires were filled out individually in a suitable environment, free from distractions. The questionnaire completion process lasted approximately 15 min. All question-naires collected were included in this study.

The research included all students from the selected grades, without any exclusion based on their culture, language, religion, race, disability, sexual orientation, ethnicity, gender, or age.

The research was approved by the Bioethics Committee of the University of Burgos, with reference number UBU 032.2/2021, adhering to all requirements outlined in the 1975 Declaration of Helsinki.

### 2.3. Assessments

Three scales with good psychometric properties, translated and validated in the Spanish population, were used for data collection.

The Academic Success Attribution Questionnaire [35,36] is a scale composed of 12 items grouped around 3 factors or dimensions: internal controllable attributions (e.g., “I pass because I work hard in class”, “I pass because I spend a lot of time preparing for exams”) related to effort, internal uncontrollable attributions (e.g., “I pass because I am very intelligent”, “I pass because I have a calm character and do not get nervous in exams”) related to ability, and external attributions (e.g., “I pass because teachers make exams easy”, “I pass because I am lucky”) related to luck and difficulty. These three dimensions correspond, respectively, to loci of controllability, stability, and causality [36,37].

Students responded to the root “I pass because…” using a Likert-type scale scoring from 1 to 5, with 1 not agreeing at all and 5 strongly agreeing.

Confirmatory factor analysis (CFA) showed a good fit of the data (χ^2^ (*p* < 0.001); χ^2^/df = 2.49; RMSEA = 0.05; SRMR = 0.043; CFI = 0.96; TLI = 0.95). The Cronbach’s alphas obtained were 0.744, 0.781, and 0.734, respectively, and indicated adequate internal consistency.

The results of the exploratory and confirmatory factor analysis revealed the adequate factor structure, internal consistency, and validity of the instrument. In addition, the ASAQ is invariant with respect to gender.

The present study takes the internal controllable attributions factor regarding aca-demic success (Cronbach’s alpha 0.744), related to effort, as the independent variable of the mediation model.

The Exams-Related Emotions Scale (EES) [35,36] was created to measure the emotions experienced by students at different times (before, during, and after) when taking an exam or test [38,39]. It consists of 31 items grouped into 3 factors: negative emotions (12 items) collects information about negative emotions experienced related to exams, such as hopelessness or anger (e.g., “Before the exam I get depressed because I feel that I do not have much hope of passing the exam”, “During the exam I get angry”, “After the exam I feel ashamed”), positive emotions (12 items) collects information about positive emotions experienced related to exams, such as hope and pride (e.g., “Before the exam I am so proud of how I prepared that I want to start the exam right away”, “After the exam I am bursting with enthusiasm”), and anxiety (7 items) collects information about experienced test anxiety (e.g., “At the start of the exam my heart starts to race”, “Before the exam I get so nervous that I wish I could miss the exam”).

Students responded to the items “Before, during or after the exam…” using a Likert-type scale from 1 to 5, with 1 being never and 5 always.

Confirmatory factor analysis (CFA) showed a good fit of the data (χ^2^ (*p* < 0.001); χ^2^/df = 1.911; RMSEA = 0.058; SRMR = 0.063; CFI = 0.913; TLI = 0.904). The Cronbach’s alphas obtained were 0.915, 0.892, and 0.866, respectively, indicating good internal consistency.

The results of the exploratory and confirmatory factor analysis revealed the adequate fac-tor structure, internal consistency, and validity of the instrument. In addition, the EES is invariant with respect to gender.

The present study takes the factor of positive emotions toward exams (Cronbach’s alpha 0.892) as a mediating variable in the mediation model.

The European Bullying Intervention Project Questionnaire (EBIPQ) is a scale that has been validated and translated into Spanish [30]. The instrument has demonstrated good psychometric properties in European countries and in Spain [31,32]. It is used to identify the prevalence of student involvement in bullying and categorizes it into victim, aggressor, victim and aggressor, and bystander (non-victim–non-aggressor) [33]. The questionnaire evaluates the frequency of aggressive behaviors or victimization, with the items specifically addressing various forms of bullying. Each subscale consists of 7 items, which are rated on a Likert-type scale ranging from 1 to 5. The response options include: No; Yes, once or twice; Yes, once or twice a month; Yes, about once a week; and Yes, more than once a week [40,41].

Academic Performance was assessed by calculating the average grade across all subjects in the most recent assessment. This measure serves as an objective (though not flawless) representation of the learning achievements in all subjects. In the Spanish education system, a uniform scoring system is employed across all educational levels (primary and secondary), where a final grade is assigned on a scale from 0 to 10.

The present study takes academic performance as the dependent variable in the mediation model.

### 2.4. Statistical Analysis

Firstly, a correlation analysis was conducted to examine the relationships between all variables included in the study: ICA, AP, and PE.

Secondly, the mediation analysis was performed using the PROCESS macro developed by Hayes [42] in SPSS. This macro allows for the estimation of indirect effects, standard errors, and confidence intervals using bootstrapping. The bootstrapping method enables statistical inference without relying on assumptions of normality or large sample sizes. In this study, a simple mediation model (PROCESS, model 4) was employed, and 10,000 boot-strapping samples were utilized. The significance of the mediated effects was assessed by examining whether the 95% confidence interval (CI) excluded the value of 0.

Finally, an analysis of variance (ANOVA) was conducted to assess significant differences in PE among different bullying categories. Additionally, a post-hoc test was per-formed to identify specific differences between individual groups.

## 3. Results

### 3.1. Correlation Analysis of Variables under Study

Table 1 shows the correlations of the variables involved in the study. Internal controllable attributions (ICA) are positively correlated with positive emotions (PE) and academic performance (AP). In addition, positive emotions (PE) are positively correlated with academic performance (AP). All these correlations are significant (*p* < 0.001).

### 3.2. Internal Controllable Attributions–Positive Emotions about Exams–Academic Performance Mediation Model (PROCESS, Model 4)

**Figure 2:**
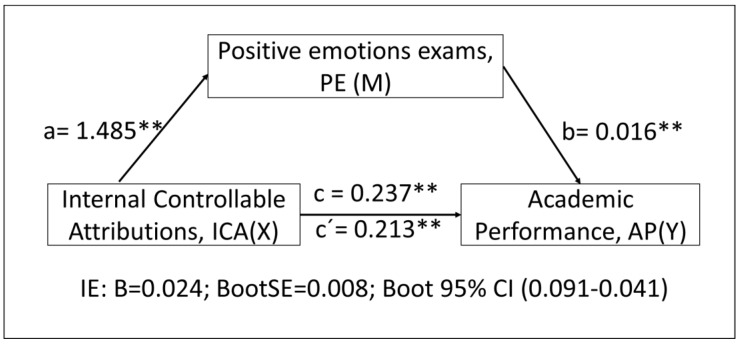
Diagram and results of simple mediation analysis: ICA-PE-AP (PROCESS 4). ** *p* < 0.001.

In Figure 2, we report the data from the simple mediation model, using as a mediating variable the EPs to the exams, as a dependent variable the AP, and as an independent variable the ICAs. The model complies with the assumptions for the application of a simple mediation analysis: significant relationships between the independent variable and the dependent variable, between the independent and mediator variable, and between the mediator and the dependent variable. In addition, the value of c is greater than that of c’.

Table 2 shows the data from the mediation analysis. The results of the regression analysis between the mediating variable PE and the independent variable ICA show a significant positive relationship (a: B = 1.1485; SE = 0.105; *p* < 0.001). The results of the multiple linear regression analysis considering ICA and PE as predictor variables show a positive significant relationship between ICA and the dependent variable AP (c’: B = 0.2134; SE = 0.016; *p* < 0.001) and between PE and AP (b: B = 0.0163; SE = 0.005; *p* = 0.004).

The total effect of the independent variable ICA on the dependent variable AP was statistically significant (c: B = 0.2375; SE = 0.014; *p* < 0.001), with the model explaining 34% of the variance of the dependent variable AP. The statistical significance of the indirect effects was demonstrated by checking that the established confidence interval (95% CI) did not contain the value 0, finding a statistically significant indirect effect (B = 0.024; BootSE = 0.008; Boot 95% CI [0.0091~0.0407]).

### 3.3. Relationship between Positive Emotions and Bullying Categories

Table 3 and Table 4 show the relationship between the PE and the different categories of bullying. Statistically significant differences are established between the bystander (42.819 ± 9.126) and victim (40.906 ± 10.171) categories (*p* = 0.049), with the bystander category demonstrating higher scores in PE. Significant differences are also found between the bystander and victim and aggressor categories (38.913 ± 10.154) (*p* = 0.001), with the bystander again demonstrating higher scores in PE, and the victim and aggressor category showing lower scores in PE.

## 4. Discussion

The aim of this work was to study the relationship between the ICA of academic success and AP mediated through the PE related to exams in students in the last few years of primary and the first few years of secondary education.

The results of the present study have shown significant relationships between all the variables analyzed, i.e., ICA, AP, and PE.

One of the fundamental theoretical perspectives when explaining AP is Weiner’s attributional theory [43], which allows us to understand how students explain their academic successes and/or failures throughout their school career. According to this approach, students’ behavior in the face of the demands of the school environment is based on interdependent episodes of both academic success and failure that are associated with positive or negative emotional responses [44,45].

Several studies show that if students experience a successful academic career, they are likely to develop a positive attributional style, attributing the causes of their success to internal and controllable causes, such as effort and ability [46]. This is why knowledge about this type of attribution facilitates an understanding of students’ motivation when faced with the requirements and tasks of the school environment. Furthermore, the idea that success in the teaching–learning process is closely linked to and can be significantly modified by variables such as emotions is pointed out [47]. Some studies agree that the rate of school success is closely related to the student’s emotional satisfaction within the school environment [48]. In line with these data, the present study shows a positive correlation between positive emotions and academic achievement.

This study shows that ICAs have a direct effect on AP, and EPs allow students to enjoy academic activity and have a greater perception of success. Some studies investigating PE, such as pride and hope, obtain results that confirm the increased perception of academic success [49,50]. According to Fredrickson [51], EPs enhance the acquisition of more personal resources for complex tasks that are present in teaching–learning processes [50]. The activation of these emotions allows students to perceive successful task performance, as opposed to negative emotions that are related to perceptions of failure [21].

A different research study also finds that AP is positively associated with intrinsic motivation and student self-efficacy and negatively associated with academic anxiety. Intrinsic motivation is an independent predictor of AP [2]. The present study corroborates these data by showing a positive correlation between internal controllable attributions for success and academic performance, both in the total and direct effects. Moreover, according to a meta-analysis, AP is associated with emotional regulation and social–emotional education has a positive impact on AP. Emotional regulation can be improved and consequently also improve AP [1,52].

The present study focuses on positive emotions as a possible mediator between internal controllable attributions to success and academic performance. Specifically, it attempts to demonstrate that ICA will produce positive emotions that in turn will positively influence academic performance.

Significantly, success through hard work, effort, and/or dedication (ICA) activates positive emotions in individuals, reinforcing self-efficacy and self-esteem [46] When students experience success through effort, they are able to increase their self-efficacy and self-esteem. Several studies have shown that success through effort is positively related to positive emotions, such as joy and satisfaction [47,48,49]. The present study supports these data by showing a positive correlation between internal controllable attributions to success and positive emotions and the mediating role of positive emotions between internal controllable attributions and academic achievement.

Finally, the second objective posed by the research was to demonstrate the existence of a relationship between the different categories of bullying and PE. Our results have shown significant differences between the categories of non-victim aggression with victim and victim with aggression. The highest scores in PE were observed in the non-victim aggression category, while the victim with aggression category showed the lowest scores in PE. Other studies have more generally observed that experiences related to bullying in some way are associated with difficulties in regulating emotions, suggesting alterations in students’ emotional intelligence, which may persist into adulthood [50,51]. However, it should be noted that those who fell under the victim with aggression category, in addition to showing the lowest scores in PE, were those who showed the lowest AP according to a previous article with the same sample of students, where statistically significant differences were also observed with respect to the categories of bullying and AP [35]. The same is true for those who fall under the non-victim aggression category, with the highest scores in both PE and AP.

Consequently, generating positive emotional environments in classrooms, away from bullying-related behaviors, favors the development of emotional competencies in students and therefore contributes to their academic development, increasing their interest in learning and improving their competencies [25].

As limitations, it should be noted that the sample was taken only from Spain and therefore it is difficult to generalize the results to the entire world population. It is important to take into account the effect of culture when attempting to extend the results to another population. Moreover, the use of self-report questionnaires may be a limitation of the research, so they should be interpreted with caution, despite being questionnaires that have demonstrated good internal consistency, validity, and reliability. Furthermore, studies in this area are scarce and there is a need for further research.

Taking into account the PE variable as a mediating variable represents a turning point when analyzing the influence of ICA on AP, so it would be interesting to collect more information by expanding the sample and data in subsequent studies. However, the scarcity of information and studies in this regard has made it difficult to compare our results with other research carried out.

## 5. Conclusions

In conclusion, it should be noted that research has indicated a close relationship be-tween AP, PE, and ICA. Controllable internal attributions of success, related to effort, facilitate the presence of positive emotions toward exams, such as pride or enthusiasm. Positive emotions positively influence academic performance. Controllable internal attributions of success show a positive relationship with academic performance, with positive emotions acting as a mediator toward academic success.

This provides us with a significant and positive predictor model for the prediction of AP as a function of ICA explained by the mediating variable of PE.

Likewise, a relationship has been established between PE and certain categories of bullying, showing that those who had lower AP [35] due to falling into a bullying category also had lower scores in PE; at the same time, bullying categories related to higher AP according to previous research showed more PE.

Therefore, we can consistently state that PE is a very powerful mediating variable to define a student’s AP, taking into account ICA, and that being involved or not in behaviors related to bullying in some way can determine the student’s PE and their AP.

Teachers and parents should take this information into account and work together to support the emotional and academic development of students.

According to the results obtained, socioemotional intervention is an important point, as emotional regulation to improve the well-being and emotional health of students, since its influence on academic performance has been demonstrated. Improving students’ emotional awareness will have a positive impact on their academic performance [1].

## Figures and Tables

**Table 1:** Results of the correlation analysis of the variables under study.

	1. ICA	2. PE	3. AP
1. ICA	-	0.514 **	0.585 **
2. PE		-	0.385 **
Mean	15.01	41.74	7.38
SD	3.32	9.61	1.35

ICA: internal controllable attributions; PE: positive emotions; AP: academic performance; SD: standard deviation. ** *p* < 0.001.

**Table 2:** Results of mediation analysis: ICA-PE-AP (PROCESS, Model 4).

Effects	Path	β	SE	*p*	
Effect ICA-PE	a	1.485	0.105	<0.0001	
Effect PE-AP	b	0.0163	0.005	0.004	
Total effect ICA-AP	c	0.2375	0.014	<0.0001	
Direct effect ICA-AP	c’	0.2134	0.016	<0.0001	
PC total effect model (F = 290.663; *p* < 0.001; R2 = 0.34)					
**Indirect Effects**		**β**	**BootSE**	**Boot 95% CI**	
				**IL**	**LL**
Total Indirect effect		0.024	0.008	0.0091	0.0407

ICA: internal controllable attributions; PE: positive emotions; AP: academic performance.

**Table 3:** Descriptive statistics: positive emotions—bullying categories.

Bullying Categories	N	Mean	SD	95% Confidence Interval for Mean	
				Lower Bound	Upper Bound
Bystander	322	42.819	9.126	41.819	43.820
Victim	139	40.906	10.171	39.200	42.612
Aggressor	20	41.700	8.826	37.569	45.830
Victim and aggressor	31	38.913	10.154	36.668	41.158

**Table 4:** Multiple group comparison of bullying categories and positive emotions.

Bullying Categories		Mean Difference	Sig	95% Confidence Interval for Mean	
				Lower Bound	Upper Bound
Bystander	Victim	1.913	0.049	0.012	3.814
	Aggressor	1.119	0.611	−3.196	5.436
	Victim and aggressor	3.906	0.001	1.578	6.234
Victim	Bystander	−1.913	0.049	−3.814	−0.012
	Aggressor	−0.793	0.728	−5.273	3.686
	Victim and aggressor	1.992	0.135	−0.625	4.611
Aggressor	Bystander	−1.119	0.611	−5.436	3.196
	Victim	0.793	0.728	−3.686	5.273
	Victim and aggressor	2.786	0.242	−1.890	7.463
Victim and aggressor	Bystander	−3.906	0.001	−6.234	−1.578
	Victim	−1.992	0.135	−4.611	0.625
	Aggressor	−2.786	0.242	−7.463	1.890
